# Study on COD and nitrogen removal efficiency of domestic sewage by hybrid carrier biofilm reactor

**DOI:** 10.1039/d1ra03286k

**Published:** 2021-08-10

**Authors:** Yuqiu Hou, Mei Liu, Xiao Tan, Siyu Hou, Ping Yang

**Affiliations:** College of Architecture and Environment, Sichuan University Chengdu 610065 China yangpinga301@163.com 740659077@qq.com 707865212@qq.com heishihe@163.com 240736328@qq.com +86 18602804508

## Abstract

A moving bed biofilm reactor (MBBR) is a kind of commonly used biological sewage treatment process. A carrier, the core of MBBR, could directly affect the treatment efficiency of MBBR. In this experiment, a hybrid carrier composed of an MBBR carrier and fluidized bed porous carrier was innovatively utilized to treat low-concentration simulated domestic sewage through an MBBR reactor to investigate the effects of different hydraulic retention times (HRT) and different carrier dose ratios on the reactor performance. The results indicated that when the volume ratio of the carrier dosage was 5% : 20% when the reactor HRT was 5 h, the removal rates of ammonia nitrogen, total nitrogen (TN) and chemical oxygen demand (COD_Cr_) were optimal, which were 96.5%, 60.9% and 91.5%, respectively. The ammonia nitrogen, total nitrogen and COD_Cr_ concentrations of the effluent were 1.04 mg L^−1^, 12.20 mg L^−1^ and 29.02 mg L^−1^, respectively. Furthermore, the total biomass concentration in the hybrid carrier biofilm reactor (HCBR) was 3790.35 mg L^−1^, which also reached the highest value. As the experiment progressed, the concentrations of protein, polysaccharide and soluble microbial products (SMP) were reduced to 7.68 mg L^−1^, 11.10 mg L^−1^ and 18.08 mg L^−1^, respectively. This was basically consistent with the results of the three-dimensional fluorescence spectrum. The results showed that the combined-carrier biofilm reactor could reduce the volumetric filling rate, improving the removal capability of organic matter and the denitrification efficiency. This study provided technical support for the composite carrier biofilm wastewater treatment technology, and also had a good prospect of application.

## Abstract 2

A combined-carrier biofilm reactor could reduce the volumetric filling rate, improving the removal capability of organic matter and the denitrification efficiency.

## Graphical abstract

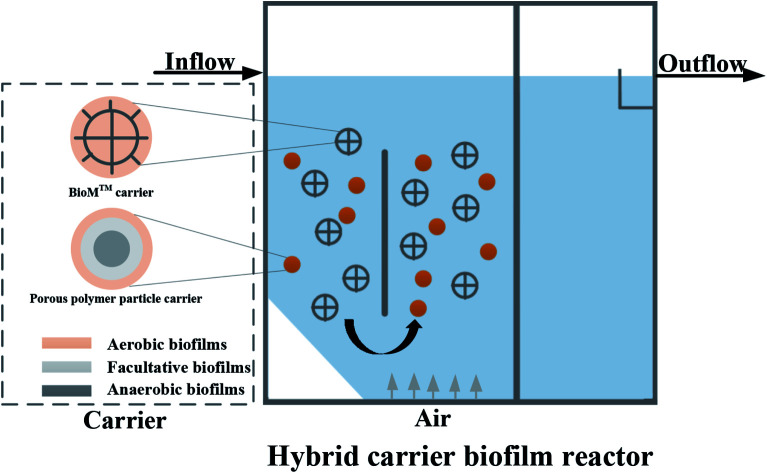

## 1. Introduction

With the development of economy and the progress of times, the sewage generated in the production and life of society has become more and more diversified. At the same time, with the continuous development of economic and ecological concepts, the discharge standards of sewage have become more and more strict.^1^ Conventional wastewater treatment methods have been challenging to meet the increasing sewage treatment requirements. At present, the most commonly used methods are activated sludge method and biofilm method. However, they have disadvantages, such as large land area, high cost and weak adaptability to changes in wastewater quality and quantity. Furthermore, the biofilm method has the disadvantages of easy clogging and regular backwashing. In order to solve the problems of these two processes,^2^ the MBBR method came into existence.

The MBBR method was developed in 1988 by the Norwegian Kaldnes company, and the Norwegian University of Science and Technology and the SINTEF research institute. The core of this process was directly adding the suspended filler with a specific gravity close to water into the aeration tank as a carrier for microbial adhesion growth. The MBBR method combines the advantages of the activated sludge method and the biofilm method, including small floor area, no need for sludge reflux or backwashing, low head loss, low power consumption, and high resistant to temperature changes and changes in sewage composition. Moreover, the treatment capacity of sewage has been improved, and the nitrification effect also has been significantly enhanced. In the past few decades, the MBBR process has been employed worldwide.^3,4^ It has been applied in treating urban domestic sewage,^3,5,6^ food industry wastewater,^7^ papermaking wastewater^8^ and landfill leachate.^9,10^ In recent years, the MBBR method was also suggested for the treatment of pharmaceutical wastewater^11^ and phosphorus removal.^12,13^

The biological fluidized bed method is a sewage treatment process developed in the early 1970s. Its principle is to use small inert porous particles, such as activated carbon, as carriers to degrade the aerobics of pollutants in wastewater. The microorganisms were immobilized on the surface of the carrier, and the carrier was fluidized to degrade the contaminants in the wastewater.^14^ Until now, biological fluidized bed reactors have been developed in various forms. They were also widely studied and applied in urban domestic sewage,^15^ industrial wastewater^14^ and landfill leachate.^16^

The integrated fixed-film activated sludge (IFAS) method integrates the hybrid biofilm process and activated sludge process to treat sewage.^17^ The bacterial richness and microbial diversity in biofilms and flocs of the IFAS system were different, which would promote high microbial diversity to achieve higher pollutant removal efficiency.

Carrier research is an important direction for the development of the biofilm process.^18^ Commonly used carrier materials can be divided into inorganic carrier materials, natural organic carrier materials, synthetic polymer carrier materials and composite carrier materials, according to their properties. With the objective to act as an effective support media for active biomass growth in a biofilm reactor, the ideal carrier material should have several desirable characteristics. Optimally, carriers should (i) have good compatibility with microorganisms and will not affect the biological activity of the microorganisms, (ii) be environmentally friendly, biodegradable, and will not cause secondary pollution, (iii) shield unfavorable external environmental conditions, provide carbon sources, nutrients and a good microenvironment, (iv) have a relatively large specific surface area; raw materials are easily available and low in price, (v) have good stability and can be used for a long time or repeatedly.^19^ The specific surface area, pore structure, mechanical strength and other factors of different carrier materials will significantly affect the immobilization process of microorganisms.^20^ A suitable carrier can improve the sewage treatment capacity. According to a previous study, in addition to polyethylene (PE), polypropylene (PP), and high-density PE (HDPE), polymers such as polyurethane (PU) and polycaprolactone (PCL) have been used as biofilm carriers in MBBRs.^21^ Among these, biofilm carriers composed of PE are often used in MBBRs because the density of PE is lower than that of other polymers.^22^ Moreover, polyethylene terephthalate (PET), produced from waste plastic bottles, can be utilized as a packing material for up-flow anaerobic sludge bed (UASB) reactor as demonstrated by M. A. EI-Khateeb.^23^ The quality of the packed UASB (P-UASB) effluent was found to be better than that of the classical UASB reactor.^23^ What is more, the down flow hanging non-woven (DHNW) reactor packed with PET has achieved excellent performance in the treatment of tanning effluents.^24^ Abu Bakar used two types of biofilm carriers to fill the MBBR to treat palm oil mill effluent. The results indicated that the hexafilter performed better than black plastic media at 50% media filling fractions (retention time of 72 h).^21^ Theoretically, the mixed-carrier biofilm reactor can simultaneously take advantage of the advantages of the two carriers and make up for each other's defects, which will greatly promote the improvement of the processing efficiency of the biofilm reactor. However, there are few research studies on mixed carrier biofilm reactors at home and abroad.

Based on this, the experiment quoted the method of IFAS, combining the MBBR carrier and the fluidized bed carrier into a combined-carrier. The simulating domestic sewage was processed through the MBBR reactor, and the influence of different HRT and carrier dosage ratios is discussed on the performance of the reactor.

## 2. Materials and methods

### 2.1 Experimental set-up

**Fig. 1:**
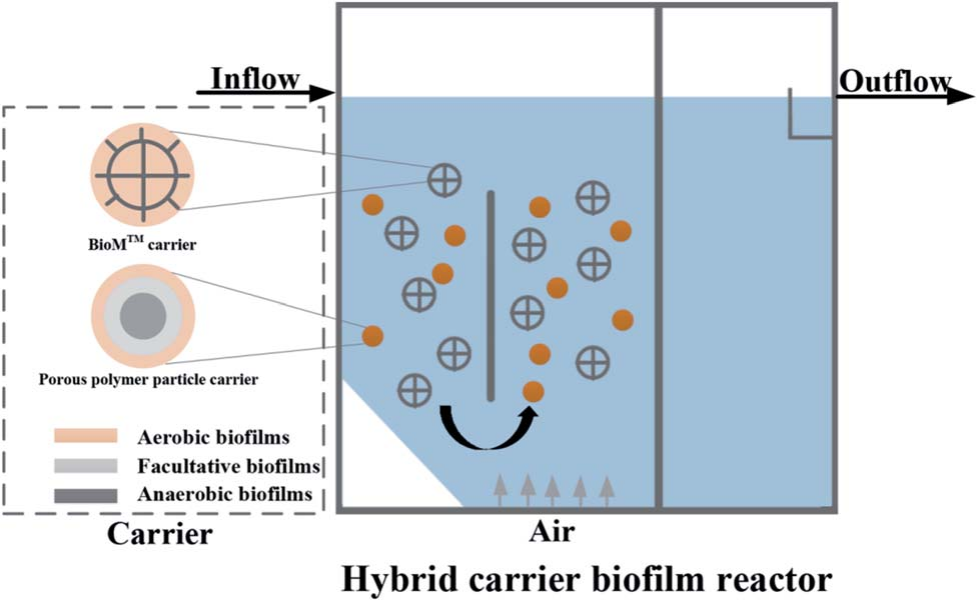
Schematic representation of the reactor.

**Fig. 2:**
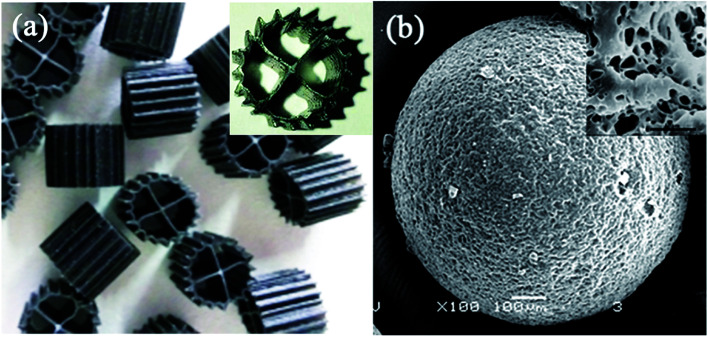
Morphologies of (a) BioM™ and (b) porous polymer particle.

The experimental device is shown in Fig. 1. The HCBR was made of plexiglass, 30 cm in length, 10 cm wide, and had an effective height of 40 cm. The reactor was divided into an aeration zone and a precipitation zone, with the effective volumes of 7.5 L and 4 L, respectively. A baffle was arranged in the middle of the aeration zone, and the aeration head was placed under the baffle to control the amount of aeration by adjusting the rotameter (connected to the air pump). Two kinds of carriers with different volume ratios were added to the pool. With the action of aeration and the flow guiding of the deflector, the hybrid carrier attached with the biofilm was counterclockwise around the deflector in the aeration zone. Most of the blank area left by the BioM™ carrier was filled by the flowing porous polymer particle carrier to ensure adequate mixing of the water, gas and solid phases in the aeration zone. Fig. 2 shows that the two carriers used in the experiment were BioM™ carrier and porous polymer particle carrier. The BioM™ carrier was produced from Dalian Yudu Environmental Engineering Technology Co. Ltd, and the porous polymer particle carrier was developed by the laboratory. The main parameters of the two carriers are shown in Table 1.

**Table 1:** The main parameters of the two carriers

Parameters	BioM™ carrier	Parameters	Porous polymer particle carrier
Type	WD-F10-4	Dry particle size (mm)	0.45–0.90
Size (mm)	*Φ*10 × 10	Skeleton density (kg m^−3^)	1320.00
Surface area (m^2^ m^−3^)	1200.00	Wet bulk density (kg m^−3^)	1010.00
Packing number (m^−3^)	495 000	Vacancy capacity (mL g^−1^)	0.301
Packing density (kg m^−3^)	125.00	Wet surface area (m^2^ m^−3^)	5357.00
Pre-film density (g cm^−3^)	0.96–0.98		
Post-film density (g cm^−3^)	1.00		

### 2.2 Experimental wastewater

The experimental wastewater was artificially prepared to simulate domestic sewage. The main components were anhydrous glucose, sodium bicarbonate, ammonium chloride, potassium dihydrogen phosphate and nutrient solution. Among them, anhydrous glucose was used to provide COD_Cr_, ammonium chloride was used to provide the nitrogen source, potassium dihydrogen phosphate was used to provide the phosphorus source, nutrient solution was used to provide other trace elements, and sodium bicarbonate was used to adjust the pH in the simulated sewage. The influent COD_Cr_ was about 350 mg L^−1^, the NH_4_^+^–N and TN concentrations were about 30 mg L^−1^, the TP concentration was about 5 mg L^−1^, and the pH was about 8.44. The composition of the nutrient solution (both of which is 0.50 mL of nutrient solution per L of synthetic wastewater) is shown in Table 2.

**Table 2:** Trace element of the nutrient solution

Nutrient solution A	Concentration (g L^−1^)	Nutrient solution B	Concentration (g L^−1^)
FeSO_4_·7H_2_O	6.985	NiCl_2_·6H_2_O	0.250
MnSO_4_·H_2_O	1.067	(NH_4_)_6_Mo_7_O_24_·4H_2_O	0.125
ZnSO_4_·7H_2_O	0.263	H_3_BO_7_	0.125
CoSO_4_·7H_2_O	0.443	CuCl_2_·2H_2_O	0.075

### 2.3 Analysis and determination methods

The water quality indexes were measured by the Chinese State Environmental Protection Administration (SEPA) standard methods. Among them, COD_Cr_ was measured by rapid digestion spectrophotometry; NH_4_^+^–N was measured by Nessler's reagent spectrophotometry; and TN was measured by alkaline potassium persulfate digestion ultraviolet spectrophotometry. The polysaccharide concentration was measured by the fluorenone–sulfuric acid method, and the protein concentration was measured by the phenol reagent method (Lowry method). The carrier attachment biomass was measured by the lye dissolution spalling method and the microscopic test diameter density calculation method. MLSS was measured by gravimetric method.

The measurement of the attached biomass on the BioM™ carrier was measured by the lye dissolution spalling method.^25^ Five randomly selected BioM™ carriers were charged into the reactor, dried at 105 °C to a constant weight, and then weighed. Then, the carriers were placed in a 0.1 mol L^−1^ NaOH solution and heated to 60 °C for 20 minutes. The degree of bonding between the biofilm and the carrier surface was greatly reduced, and the biofilm on the carrier was peeled off mechanically to remove water. The carriers were washed and dried at 105 °C to a constant weight, and then weighed. The total weight of the attached biomass on the carriers was calculated and converted to the biomass concentration of the attached biofilm on the BioM™ carrier throughout the reactor.

The amount of attached biomass on the porous polymer particle carrier was determined by the microscopic test diameter density calculation method. Boaventura *et al.*^26^ and Coelhoso *et al.*^27^ found that the density of the biofilm attached to the biofilm carrier was related to its thickness. When the thickness of the biofilm was less than a certain value, the density of the biofilm decreased linearly with the increase of the thickness. Moreover, when the film thickness increased to a certain value, the density of the biofilm no longer changed with the increase of the film thickness. The biofilm density *ρ* fitted by Boaventura *et al.*^28^ through experimental data was calculated by the following eqn.

1 *ρ* (mg cm^−3^) = 104.3 − 0.224*L* *L* < 622 μm

2 *ρ* (mg cm^−3^) = 26.9 *L* > 622 μm

The thickness *L* of the biofilm adhered to the porous polymer particle carrier was measured by referring to the experimental method of Pan *et al.*^29^ The irregular porous polymer particle carrier was regarded as an ellipsoid, and the long axis *a* of the ellipsoid was measured by an optical microscope. The short axis *b* was converted into the equivalent sphere diameter *d* = (a·b^2^)^1/3^. In each test, 100 carrier particles were randomly selected from the reactor for testing. The average diameter of Sauter was used to represent the average diameter of 100 carrier particles De = ∑*d*^3^/(∑*d*^2^). In addition, 100 optical carrier particles were selected in the same way to test the average diameter Dc = 1351.66 μm, the volume *V*_0_ = 1.293 mm^3^, and the biofilm thickness *L* is (De − Dc)/2. After the biofilm density *ρ* was obtained according to the thickness of the biofilm, the biofilm volume was determined: 
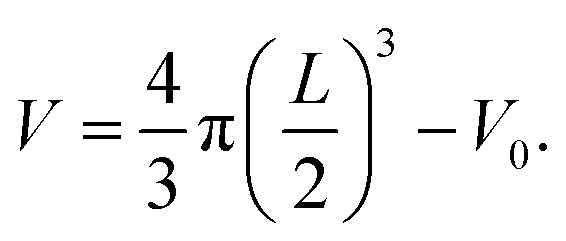
 Finally, the biomass concentration of the biofilm was *X* = *ρV*.

### 2.4 Experimental start-up

The hybrid carrier biofilm reactor used in this experiment was from another experiment that has treated high-salt wastewater in the same laboratory. After using the simulated domestic sewage to recover the reactor in a low-salt state for a period of time, the biofilm was replenished by using a quick biofilm culturing method. The aerobic-activated sludge inoculated with the biofilm was from the sedimentation tank of Baijia Sewage Treatment Plant in Shuangliu County, Chengdu.

## 3. Results and discussion

### 3.1 HCBR for treatment efficiency of simulated domestic sewage

To investigate the effects of different hydraulic retention times (HRT) and different carrier dose ratios on the reactor performance, the simulated domestic sewage in this experiment was treated by adjusting the ratio of the two kinds of carriers at different operating conditions. The specific operating conditions of our experiment are shown in Table 3. The experiment was divided into 12 working conditions according to the different HRT and carrier dosage ratios: in the first part P1, the HRT of the reactor operation was 11 h, 8 h, 5 h and 2.5 h, respectively. The ratio of the porous polymer particle carrier to the BioM™ carrier was 2% : 20%, and the specific dosages were 0.15 L and 1.5 L. In the second part P2, the HRT of the reactor operation was 2.5 h, 5 h, 8 h and 11 h, respectively. The ratio of the porous polymer particle carrier to the BioM™ carrier was 5% : 20%, and the specific dosages were 0.375 L and 1.5 L. In the third part P3, the HRT of the reactor operation was 11 h, 8 h, 5 h and 2.5 h, respectively. The ratio of the porous polymer particle carrier to the BioM™ carrier was 8% : 20%, and the specific dosages were 0.6 L and 1.5 L. The performance of the HCBR for the treatment of synthetic simulated domestic sewage was studied. The optimum carrier dosing ratio of the HCBR was explored under the different pollutant removal loads. Furthermore, the changes of biomass and SMP in the reactor under different working conditions were investigated.

**Table 3:** Experimental parameters of operating conditions

	Carrier ratio	HRT (h)	Volume loading	
			kg COD (m^3^ d)^−1^	kg NH_3_–N (m^3^ d)^−1^
P1	2% : 20%	11	0.87	0.076
		8	1.2	0.105
		5	1.92	0.168
		2.5	3.84	0.42
P2	5% : 20%	2.5	3.84	0.42
		5	1.92	0.168
		8	1.2	0.105
		11	0.87	0.076
P3	8% : 20%	11	0.87	0.076
		8	1.2	0.105
		5	1.92	0.168
		2.5	3.84	0.42

#### 3.1.1 Removal of COD_Cr_ in the HCBR

The removal of COD is related to biodegradable carbon.^30^ The COD in the simulated domestic sewage of this experiment was all provided by glucose, and the microbial degradation reaction is as follows:^31^

3 C_6_H_12_O_6_ + 6O_2_ → 6CO_2_ + 6H_2_O

**Fig. 3:**
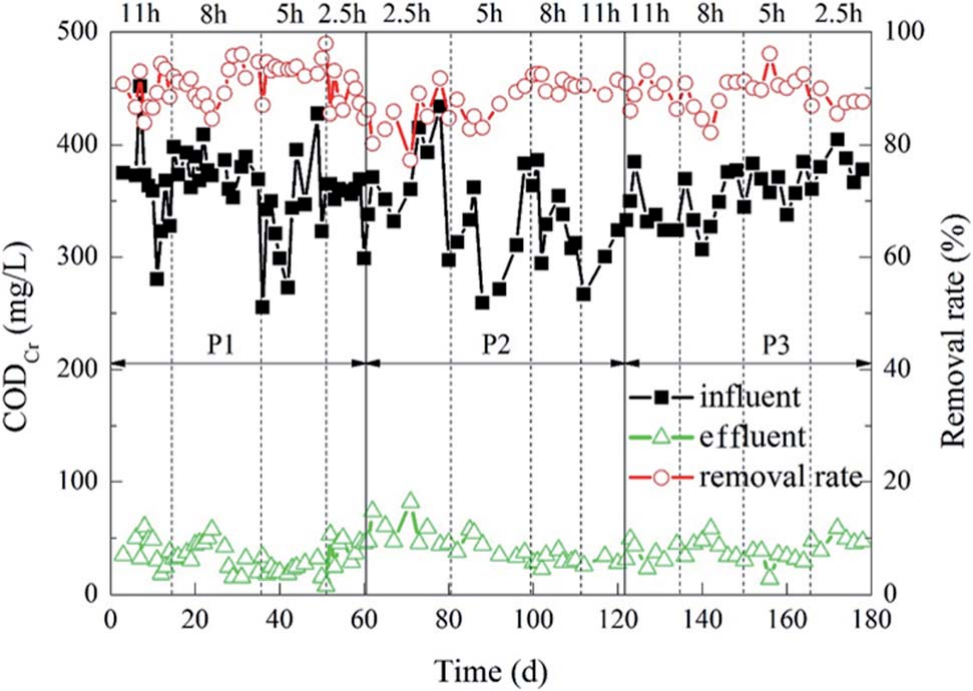
Dynamic changes of HCBR for COD_Cr_ removal (P1, P2 and P3 represented the operations in which the ratios of the porous polymer particle carrier to the BioM™ carrier were 2% : 20%, 5% : 20% and 8% : 20%, respectively).

The removal of COD_Cr_ during the experiment is shown in Fig. 3. Under different carrier ratios, the COD_Cr_ removal effect of the reactor was almost flat. When the HRT were 5 h, 8 h and 11 h, the average effluent COD_Cr_ of the reactor was about 30 mg L^−1^, and the average COD_Cr_ removal rate was about 90%. When the HRT was 2.5 h, the COD_Cr_ removal rate of the reactor decreased slightly, and the effluent COD_Cr_ concentration increased slightly. The average concentration was 45.2 mg L^−1^, and the average removal rate was about 87%. In the 12 working conditions of the experiment, when the carrier ratio was 2% : 20% and the HRT was 5 h, the average effluent COD_Cr_ of the reactor was 22.89 mg L^−1^ and the average removal rate of COD_Cr_ was 93%. That was optimal processing efficiency.

The results indicated that the carrier ratio had little effect on the COD_Cr_ removal effect of the HCBR. The removal rate of COD_Cr_ was positively correlated with the change of HRT.^32^ This change may due to the fact that different HRTs would cause the F/M in the reactor to be different.^33^ The shorter the HRT was, the larger the F/M was. Meanwhile, the carrier provides a stable growth environment for microorganisms, thus improving the degradation effect of the reactor.^34^ However, when the loading was so high that the microorganisms in the reactor could not degrade the organic matter in time, this resulted in a decrease of the COD_Cr_ removal rate. When the HRT was prolonged, the microorganisms attached to the carriers would have enough time to contact the organic matter and remove it.^32^ However, in this experiment, the change of the COD_Cr_ removal rate with HRT changes was not prominent, which indicated that the HCBR had a wide load range and strong impact resistance.

#### 3.1.2 Removal of NH_4_^+^–N in the HCBR

Microorganisms degrade NH_4_^+^–N through nitrification, and the reactions are as follows:^35^

4 2NH_4_^+^ + 3O_2_ → 2NO_2_− + 2H_2_O + 4H^+^

5 2NO_2_^−^ + O_2_ → 2NO_3_^−^

**Fig. 4:**
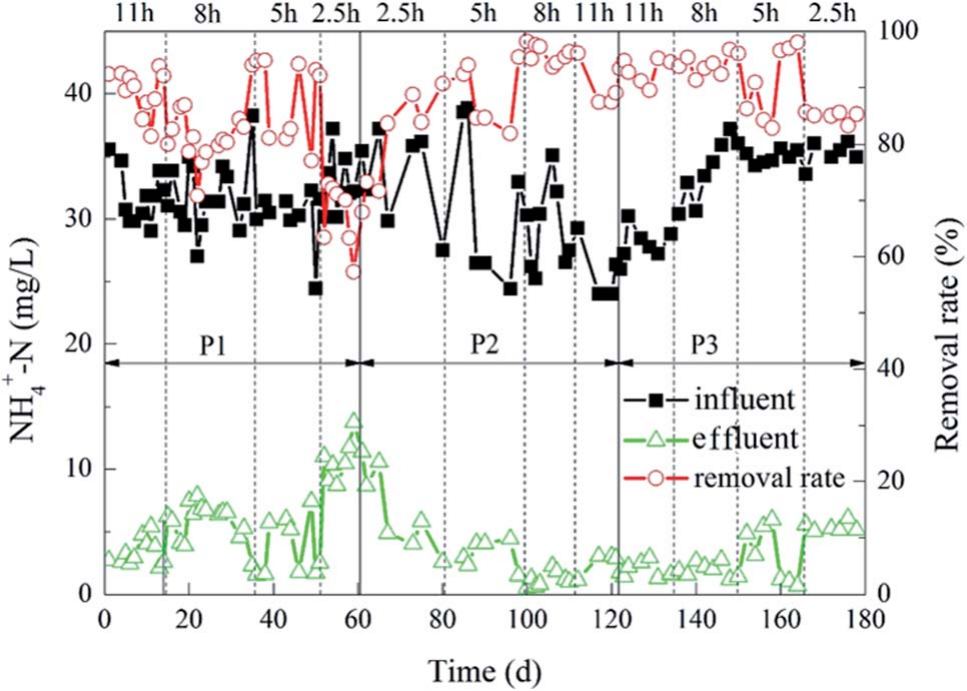
Dynamic changes of HCBR for the ammonia–nitrogen removal.

The degradation of NH_4_^+^–N during the experiment is shown in Fig. 4. When the ratios of the carrier dosage were 5% : 20% and 8% : 20%, the removal rate of NH_4_^+^–N in the whole process of the reactor was higher than the removal rate when the dosage ratio was 2% : 20%. Furthermore, when the ratios of the carrier dosage were 5% : 20% and 8% : 20%, the removal rates of NH_4_^+^–N were not significantly different from each other. After the reactor stabilized, when HRT was 5 h, the removal rate of NH_4_^+^–N was optimal with the dose ratios of 5% : 20% and 8% : 20%. The average NH_4_^+^–N effluent was 1.10 mg L^−1^ and 0.99 mg L^−1^, and the removal rates were 96% and 97%, respectively. When the HRT was 2.5 h, the effluent NH_4_^+^–N removal rate was less than 90%. Moreover, when the carrier dosage ratios were 5% : 20% and 8% : 20%, the effluent NH_4_^+^–N concentration was 5 mg L^−1^ ± 1 mg L^−1^. When the HRT were 8 h and 11 h, the removal rate of NH_4_^+^–N in the reactor was above 90%, which was slightly lower than the removal rate of 5 h HRT.

The results indicated that different carrier dosage ratios and different HRT both had effects on the removal of NH_4_^+^–N. The reason may be that there was less space for microorganisms to attach on the carrier to generate biofilm in the reactor when the carrier dosage ratio is 2% : 20%, resulting in less microbial biomass in the reactor and low removal rate of NH_4_^+^–N. However, when the ratio of the carrier was 8% : 20%, the removal rates of NH_4_^+^–N was not higher than the removal rates when the carrier dosage ratio was 5% : 20%. It may be because the excessive carrier filling rate also means the collision between the carriers frequently increased, resulting in an increase in the rate of biofilm desorption on the surface of the carrier.^36^ Therefore, the amounts of microorganisms in the reactor would not increase greatly with the increase of the carrier ratio, and the removal rate of NH_4_^+^–N. The change of HRT had a great influence on the efficiency of NH_4_^+^–N removal in the reactor.^37^ The removal rate of NH_4_^+^–N would decrease rapidly by shortening the HRT,^38^ and the removal rate of NH_4_^+^–N would increase significantly by increasing the HRT. The reason may be that the excessive HRT leads to a decrease in the organic load in the reactor, which in turn reduced the microbial metabolic activity^39^ and the removal rate of NH_4_^+^–N. At the same time, the increase of the amount of the porous carrier was beneficial to the removal of NH_4_^+^–N, while the effect was not very obvious.

#### 3.1.3 Removal of TN in the HCBR

The biofilm is attached to the carriers, and the oxygen concentration gradually decreases during the dissolved oxygen mass transfer process. Three areas are formed inside the biofilm: anaerobic zone, facultative anaerobic zone and aerobic zone. The aerobic zone is where the nitrification reaction takes place (eqn (4) and (5)), and the facultative anaerobic zone and anaerobic zone are where the denitrification reaction takes place, thus realizing the removal of TN. The denitrification reaction is as follows:^40^

6 6NO_3_^−^ + 2CH_3_OH → 6NO_2_^−^ + 2CO_2_ + 4H_2_O

7 6NO_2_^−^ + 3CH_3_OH → 3N_2_ + 3CO_2_ + 3H_2_O + 6OH^−^

**Fig. 5:**
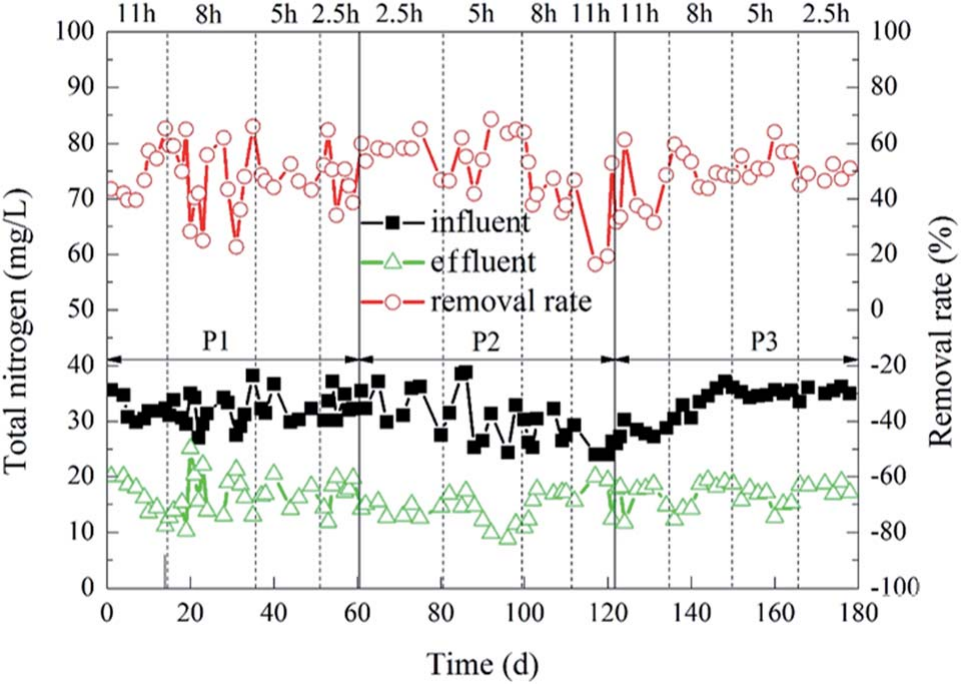
Dynamic change of HCBR for total nitrogen removal.

The degradation of TN during the experiment is shown in Fig. 5. When the HRT was 11 h or 8 h, 2% : 20% of the carrier dosage ratio was more superior. The average TN removal rate at this time was 50%. In contrast, when the HRT was 2.5 h or 5 h, 5% : 20% and 8% : 20% of the carrier dosage ratios were better. Furthermore, 5% : 20% of the carrier dosage ratio was better than 8% : 20% of the carrier dosage ratio. When the carrier dosage ratio was 5% : 20% and the HRT was 5 h, the TN removal rate of the reactor was optimal. The average effluent TN concentration was 12.20 mg L^−1^, and the average removal rate was 61%. The possible reason for this difference was that the amounts of microorganisms in the reactor was small when the carrier dosage ratio was 2% : 20%. Furthermore, when the organic load was low, fewer microorganisms were just able to digest organic matter over a long HRT. However, it was difficult for the microorganisms to completely degrade organic matter in the reactor when the organic load was high, so the TN removal rate was not very good in this situation. However, when the carrier dosage ratios were 5% : 20% and 8% : 20%, the amount of microorganisms in the reactor was larger, and the ability to decompose organic matter was stronger. Furthermore, the carrier dosage ratio at 5% : 20% was better than 8% : 20%. It was probable that when the ratio of the carrier dosage was 8% : 20%, the carriers in the reactor were more likely to collide with each other. This affected the adhesion of the biofilm outside the carrier,^36^ which in turn affected the removal rate of TN in the reactor. The change of HRT also had a great influence on the removal rate of TN.^41^ When the HRT was too short, the nitrogen in the sewage and the microorganisms on the biofilm would be sufficiently contacted, which also increased the organic load in the reactor and weakened the stability of the biofilm.^42^ However, when the HRT was too long, the amount of organic matter in the reactor would decrease,^39^ and the microbial metabolic activity would decrease, which also affected the removal rate of TN.

### 3.2 Changes of biomass in the HCBR

#### 3.2.1 Changes in biomass attached to the BioM™ carrier

**Fig. 6:**
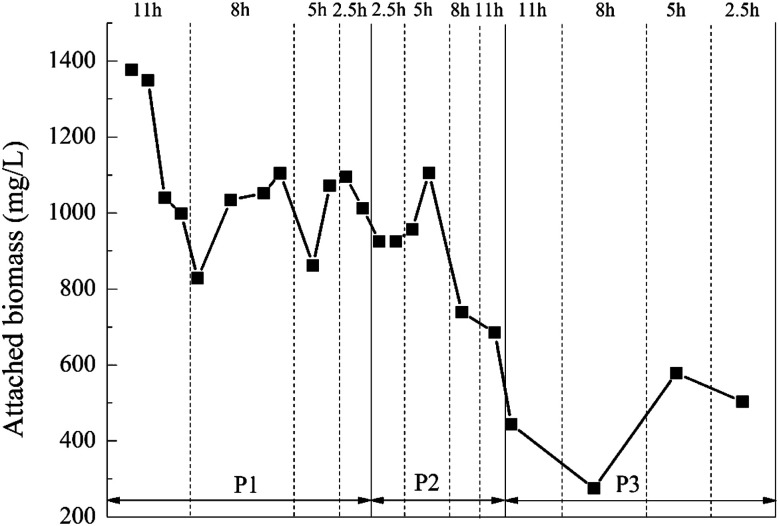
Dynamic change of the attached biomass concentration in the BioM™ carrier.

The changes in the biomass of the biofilm attached to the BioM™ carrier during the experiment is shown in Fig. 6. It can be found that the biofilm attachment growth on the BioM™ carrier is more suitable when the reactor had HRT of 5 h. At the dosage ratio of 5% : 20% and 8% : 20%, the biofilm attached to the BioM™ carrier grew best when HRT was 5 h, and the attached biomass was the highest. That was because when the HRT was 2.5 h, it was too short and increased the hydraulic shear.^43^ However, too long HRT increased the difficulty of microbes attaching to the carriers.^44^ At the dosage ratio of 8% : 20%, there was a significant decrease in the amount of attached biomass on the BioM™ carrier. It was not difficult to find that as the dosage ratio of the carrier increased, *i.e.*, the dosage of the porous polymer particle carrier was increased, the biomass attached to the BioM™ carrier tended to decrease. That was because the total biomass that the reactor can carry was not infinite.^45^ When the dosage of the porous polymer particle carrier reached 0.6 L, it dominated the reactor and continuously squeezed the living space of the attached microorganism on the BioM™ carrier.

#### 3.2.2 Changes in attached biomass on porous polymer particle carriers

It has been observed in the experiment that when the carrier dosage ratio was 2% : 20%, the porous polymer particle carrier could not guarantee a stable fluidization state for a long time under the aeration flow rate of 1.5 L min^−1^, and there were few organisms on the carrier. Only the attached biofilm thickness of the porous polymer particle carrier was determined in the experiment when the carrier dosage ratios were 5% : 20% and 8% : 20%.

The change in the attached biomass on the porous polymer particle carrier under different operating conditions of the reactor is shown in Table 4. When the ratio of the carrier dosage was 5% : 20%, the biomass attached to the porous polymer particle carrier was more than 8% : 20% carrier dosage ratio under each HRT. The reason for this phenomenon may be due to excessive carrier. The probability of collision with each other during the movement of the reactor would increase, and the microorganisms attached to the carrier would fall off during the collision.^36^ At the same time, with the shortening of the HRT, the biomass attached to the porous carrier under both dosing ratios tended to increase. This might be owing to the HRT shortened so that the organic load increased, which in turn led to a faster growth of biomass.^46^

**Table 4:** Change of the attached biomass concentration in porous polymer particle carriers

	Dosage	2.5 h	5 h	8 h	11 h
Particle size (μm)	0.375 L	1454.59	1451.27	1436.57	1422.07
	0.6 L	1446.25	1445.64	1428.40	1411.73
Film thickness (μm)	0.375 L	51.46	49.81	42.46	35.20
	0.6 L	47.30	46.99	38.37	30.04
Density (mg cm^−3^)	0.375 L	97.89	98.10	99.01	99.92
	0.6 L	98.41	98.45	99.52	100.56
Biomass (mg L^−1^)	0.375 L	1682.76	1628.13	1385.97	1147.64
	0.6 L	1545.43	1535.39	1251.72	978.18

**Fig. 7:**
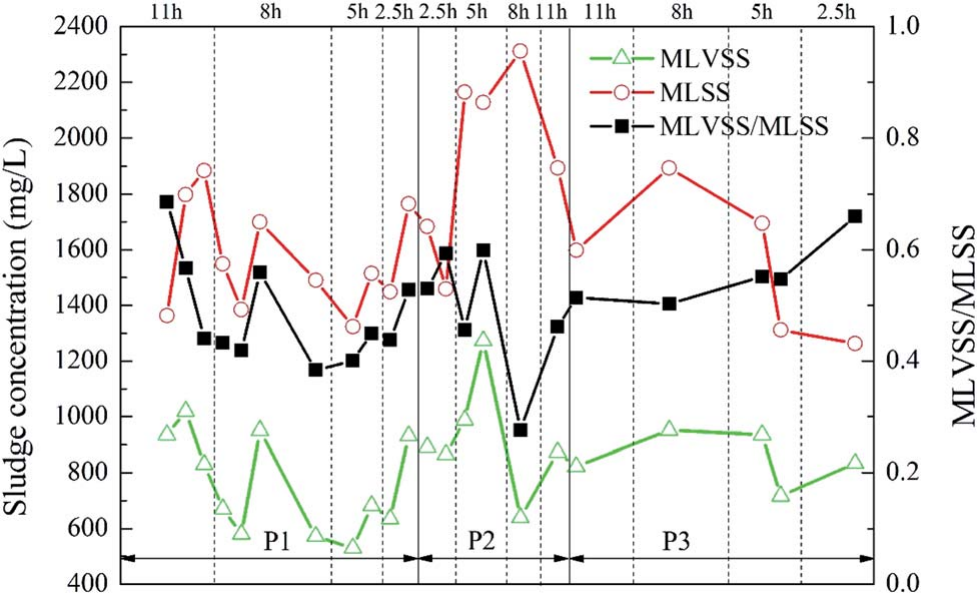
Dynamic changes of the sludge concentration in anaerobic granular sludge.

Comparing Fig. 7 with Table 4, the attached biomass of the porous polymer particle carrier was generally higher than the BioM™ carrier. When the HRT was 2.5 h, the organic load was high and the attached biomass on the BioM™ carrier was affected and reduced, while the attached biomass on the porous polymer carrier still maintained an increasing trend. This indicated that the porous polymer particle carrier had a better microbial immobilization effect than the BioM™ carrier. The reason is that the porous polymer particle carrier has a porous structure and good permeability. The microbial flora could be simultaneously attached to the surface of the carrier and the internal pores. This porous structure can significantly increase the degree of microbial aggregation, and facilitate both the metabolism of microorganisms and the diffusion of metabolites, thereby increasing the immobilization effect of the carrier on microorganisms.^47^

#### 3.2.3 Variation of suspended sludge concentration

During the operation of the reactor, a part of the porous polymer particle carrier was naturally deposited at the bottom of the aeration zone and the sedimentation zone. Furthermore, a part of the suspended sludge was intercepted by these carriers entering the sedimentation zone from the aeration zone by filtering, so the activated sludge in the reactor can be maintained in a certain amount. In the experiment, the changes of parameters, such as MLSS, MLVSS, MLVSS/MLSS, were observed by gravimetric method.

The variation of sludge concentration under different working conditions is shown in Fig. 7. As the ratio of the carrier dosage increased, the sludge concentration in the reactor first increased and then decreased. When the carrier ratio was 5% : 20% and the HRT was 5 h, MLVSS reached highest, which was 1273.33 mg L^−1^. Under different HRT, the MLVSS/MLSS value showed a low trend in the middle. That is, MLVSS/MLSS was higher when the HRT was longer or shorter. However, the activity of the suspended sludge in the reactor was generally low, and the MLVSS/MLSS was kept at about 0.5. Since this experiment mainly studied the attachment of biofilm on the carrier, the change of the concentration of the suspended sludge in the reactor was not considered as the key analysis.

#### 3.2.4 Changes in total biomass

The biomass concentration of the BioM™ carrier in the reactor and the porous polymer particle carrier under different operating conditions (the carrier dosage ratio was 2% : 20%, the biomass concentration was recorded as 0), and the mixture volatile sludge concentration (the sum of MLVSS) was used as the total biomass concentration of the reactor during this period and analyzed.

The results are shown in Table 5. When the ratios of carrier dosage were 5% : 20% and 8% : 20%, the total biomass in the reactor increased with the HRT approaching 5 h. The total biomass in the reactor reached the highest when the HRT reached 5 h. It was speculated that this phenomenon was due to the increasing hydraulic shear caused by too short HRT.^43^ Hydraulic shearing had a great influence on the peeling of biofilms.^48^ Excessive hydraulic shearing could increase the difficulty of attaching and fixing microorganisms to the carrier.^44^ However, if the HRT was too long, the organic load in the reactor would decrease, which may affect the increase of the amount of biomass attached. Moreover, the biomass at 5% : 20% of the carrier dosage ratio was more than 8% : 20%, indicating that the growth of microorganisms in the reactor would be affected if the carrier dosage ratio was too high.

**Table 5:** The total biomass concentration changes of the HCBR

	Carrier dosage ratio	2.5 h	5 h	8 h	11 h
Biomass (mg L^−1^)	2% : 20%	1837.40	1572.72	1697.95	2119.61
	5% : 20%	3473.04	3790.35	2763.34	2706.51
	8% : 20%	2882.02	2939.77	2748.79	2243.67

When the carrier dosage ratio was 2% : 20%, the change of total biomass in the reactor was exactly opposite. When the HRT was 2.5 h or 11 h, the biomass in the reactor was significantly higher. The reason for this phenomenon was presumed to be that the carrier dosing ratio was too low, and the total biomass was mainly derived from the biomass attached to the BioM™ carrier.

### 3.3 Changes in SMP in the HCBR

Soluble microbial products (SMP) were one of the important components of dissolved COD_Cr_ in the effluent of the bioreactor.^49^ It was the dissolved substances released to the outside during the matrix decomposition process of microorganisms, the process of degrading pollutants and its own metabolism, as well as attenuation, death, endogenous respiration process or response to environmental stress. SMP can be divided into two types according to the generation method:^50^ UAP and BAP, which were associated with substrate metabolism and biomass growth and associated with biomass decay, respectively. They were mainly produced by the microbial matrix decomposition process (UAP) and endogenous respiration process (BAP).^51^ Generally, the growth process, maintaining concentration balance, hunger stimulation, lack of matrix in the environment, impact load, substrate stimulation and relieving environmental stress were all the main ways of SMP production.^49^ The important factors affecting the production of SMP were microbial growth, hunger stimulation and endogenous metabolism. The detection and analysis of the SMP in the effluent of the reactor helped to understand the growth state of the sewage treatment microorganisms in the HCBR.

The main components of SMP mainly included proteins, polysaccharides, humic acids, nucleic acids, antibiotics, organic acids, and others.^52^ Most of them were fluorescent substances. Proteins and polysaccharides were the main ingredients that were ubiquitous in a variety of different situations.^53^ In this experiment, proteins, polysaccharides and the characteristic peaks of their three-dimensional fluorescence spectra were used simultaneously as indicators to indicate SMP.

#### 3.3.1 Changes in protein and polysaccharide concentrations

**Fig. 8:**
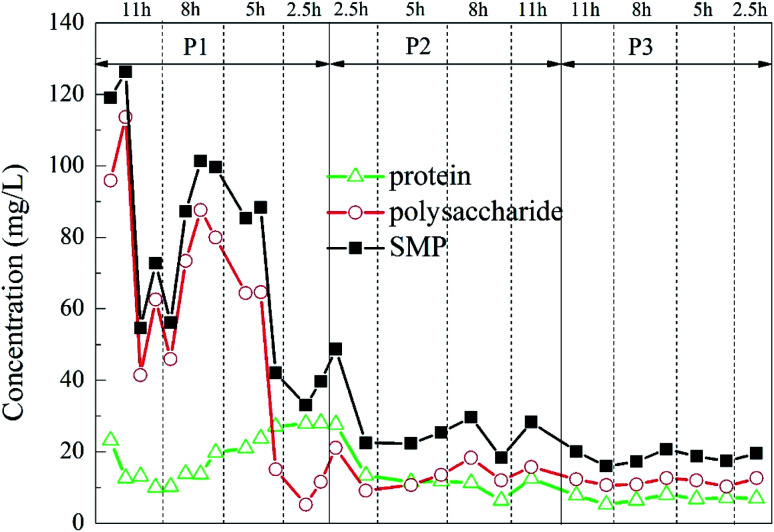
Dynamic changes of the protein, polysaccharide and SMP concentrations in the HCBR effluent.

In this experiment, the protein concentration and polysaccharide concentration in 12 kinds of operations with different carrier dosages and different HRTs were tested. The degradation of the protein and polysaccharide is shown in Fig. 8. The concentration of SMP in the figure was expressed as the sum of the protein and polysaccharide concentrations in the same period. At the beginning of the experiment, the concentrations of protein, polysaccharide and SMP in the effluent of the reactor were high, and the average concentrations were about 14.40 mg L^−1^, 71.71 mg L^−1^ and 86.11 mg L^−1^, respectively. As the experiment progressed, the concentrations of protein, polysaccharide and SMP gradually decreased, and the average effluent concentrations were 7.68 mg L^−1^, 11.10 mg L^−1^ and 18.08 mg L^−1^, respectively.

Secretion of SMP was found to increase during stress conditions,^54^ and excessive SMP would have a negative impact on the reaction.^55^ The SMP concentration of the initial effluent changed greatly, presumably due to the effect of the previous high-salt wastewater degradation experiment on the microorganisms in the reactor before the start of the experiment. In the middle and late stages of the experiment, the SMP concentration in the effluent of the reactor was greatly reduced and it became relatively stable with the change of HRT, indicating that the microorganisms in the reactor have adapted to the influence of the change of the hydraulic conditions, and it could adapt well under each HRT. With the increase of the ratio of carrier dosage, the SMP concentration in the effluent decreased slightly in a stepwise manner, demonstrating that the addition of the porous polymer carrier had less influence on the microorganisms in the reactor.

#### 3.3.2 Changes in the three-dimensional fluorescence spectrum

Most of the dissolved organic substances contained in the sewage were fluorescent substances, such as oils, proteins, surfactants, humus, aromatic compounds and the like. By measuring the three-dimensional fluorescence spectrum (EEM) of the sample, the fluorescence characteristic peak and the change of the fluorescence intensity were analyzed to obtain the change of the concentration and structure of the fluorescent substance. On a three-dimensional fluorescence spectrum, the fluorescence intensity was expressed as a function of two variables: the excitation wavelength (EX) and the emission wavelength (EM). Each of them had a corresponding specific fluorescence center. In this experiment, the three-dimensional fluorescence spectra of the effluent under different conditions of different carrier dosages and different HRT were plotted. The spectrogram had three main fluorescent peaks (A, B and C), and the central positions were located at 210–230/290–350 nm, 320–350/410–420 nm and 270–280/310–350 nm, respectively. Among them, the A and C peaks represented proteins and polysaccharides,^56^ respectively, and B peak represented humus.^46^

**Fig. 9:**
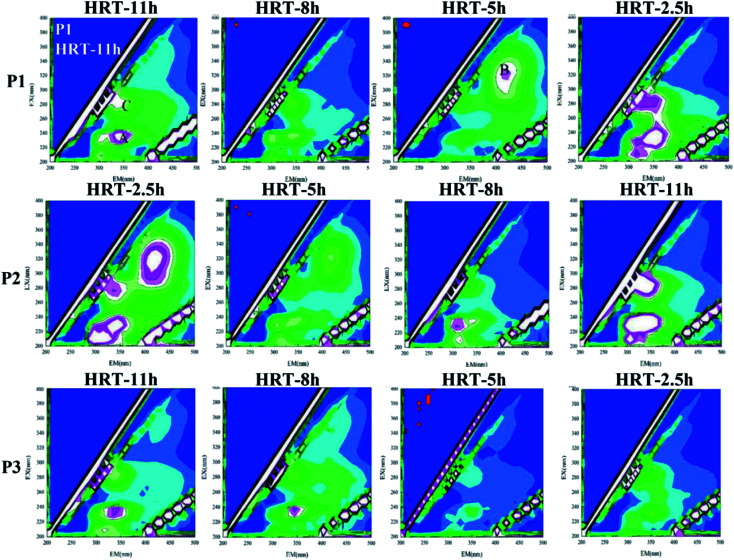
EEM spectrogram of the effluent of HCBR.

The change is shown in Fig. 9. It could be found that under different working conditions, the change of the fluorescence intensity of each characteristic peak with time is similar to the change of the sum of protein and polysaccharide in Fig. 9. In the P1 stage, *i.e*., when the carrier dosage ratio was 2% : 20%, the sum of the fluorescence intensities of the organic substances varied depending on the HRT. When the HRT was 5 h, the sum of the characteristic fluorescence intensity of each organic matter was the lowest, which indicated that the HRT of 5 h was the optimum operating condition for reducing the SMP of the water at this stage. In the P2 stage, *i.e.*, when the carrier dosage ratio was 5% : 20%, the characteristic fluorescence intensity of each organic substance in the effluent of the reactor also changed greatly with the change of the HRT. When the HRT was 8 h, the sum of the characteristic fluorescence intensities of each organic matter was the lowest, which indicated that the HRT of 8 h was the optimum operating condition for reducing the SMP of the water at this stage. In the P3 stage, *i.e.*, when the carrier dosage ratio was 8% : 20%, the characteristic fluorescence intensity of each organic matter changed little with the change of HRT, and the concentration of effluent SMP did not change substantially with the change of HRT. This indicated that at this stage, the microbial system in the reactor was relatively mature, and the load-resistance ability was greatly improved. When the HRT was 5 h, the sum of the characteristic fluorescence intensity of each organic matter was the lowest, indicating that the HRT of 5 h was the optimum operating condition for reducing the SMP of the effluent at this stage.

When the HRT was 5 h in the P1 phase and the HRT were 2.5 h and 5 h in the P2 phase, the B peak representing humus appeared in the spectrum, which was completely different from the effluent under other HRTs. It indicated that the pollutant degradation pathway of the microbial system in the reactor changed at this stage due to the change of HRT, which resulted in a large change in the proportion of components in the effluent SMP. In other cases, the sum of the fluorescence intensities of the characteristic peaks was strong, which manifested that the activity of the microorganisms was stimulated at this time, resulting in an increased secretion in the effluent SMP to alleviate the environmental stress. The sum of the characteristic fluorescence intensities of the organic matter in the P3 stage was generally small. The change with the change of the HRT was also not obvious. It implied that at this stage, the microbial system in the reactor was relatively mature. The load-resistance ability was greatly improved, and the degradation pathway of pollutants was relatively stable.

## 4. Conclusion

In this experiment, the combined-carrier biofilm reactor was used to treat domestic sewage under different HRT and carrier dosing ratios. Results indicated that when the dosage ratio of the porous polymer particle carrier to the BioM™ carrier was 5% : 20% and the HRT was 5 h, the HCBR achieved the optimal treatment efficiency on simulated domestic sewage. As the dosage of the porous polymer particle carrier increased, the attached biomass on both carriers decreased and the sum of protein and polysaccharide concentrations gradually decreased. When the dosage ratios of the carriers were 5% : 20% and 8% : 20%, the concentrations of protein and polysaccharide in the effluent of the reactor were relatively stable with the change of HRT.

## Conflicts of interest

There are no conflicts to declare.

## Supplementary Material
